# The PRINTEMPS study: protocol of a cluster-randomized controlled trial of the local promotion of a smartphone application and associated website for the prevention of suicidal behaviors in the adult general population in France

**DOI:** 10.1186/s13063-020-04464-2

**Published:** 2020-06-22

**Authors:** Coralie Gandré, Anaïs Le Jeannic, Marie-Amélie Vinet, Kathleen Turmaine, Philippe Courtet, Jean-Luc Roelandt, Guillaume Vaiva, Bruno Giraudeau, Corinne Alberti, Karine Chevreul

**Affiliations:** 1 Université de Paris, Unité UMR 1123 ECEVE, INSERM, Paris, France; 2 Health Economics Clinical Research Platform (URC Eco), AP-HP, 1 Place du Parvis Notre-Dame, 75004 Paris, France; 3 Department of Psychiatric Emergency and Acute Care, Lapeyronie Hospital, CHU Montpellier, Montpellier, France; 4 Neuropsychiatry, Epidemiological and Clinical Research, INSERM, University of Montpellier, Montpellier, France; 5 World Health Organization Collaborating Centre for Research and Training in Mental Health, Établissement Public de Santé Mentale Lille Metropole, Lille, Hellemmes France; 6 Department of Adult Psychiatry, CHU Lille, Lille, France; 7 Centre National de Ressources et Résilience pour le Psychotraumatisme (Cn2r Lille Paris), Lille, France; 8 INSERM CIC 1415, CHRU de Tours, Tours, France; 9 Tours University, Nantes University, INSERM SPHERE, U1246, Tours, France; 10 Hôpital Robert Debré, CIC-EC, Unité INSERM CIC 1426, Assistance Publique-Hôpitaux de Paris, Paris, France

**Keywords:** E-health, Mental health, Promotion, Psychological distress, Suicide, Prevention, Help-seeking, Local authorities, Intervention, General population

## Abstract

**Background:**

Suicide constitutes a cause of death which could be prevented by e-health programs accessible to the general population. Effective promotion has the potential to maximize the uptake of such programs. However, few e-health programs have been combined with promotion campaigns. The primary objective of this trial is to assess the effectiveness of a tailored promotion, at a local level, of a mobile application and website offering evidence-based content for suicide prevention (the StopBlues program), and to compare the effectiveness of two types of local promotion in terms of their impact on suicidal acts. Secondary objectives focus on the effectiveness of the promotion in terms of the intensity of utilization of the StopBlues program, help-seeking behaviors and the level of psychological impairment of program users.

**Methods/design:**

This is a three-arm, parallel-group, cluster-randomized controlled trial, with before-and-after observation. Thirty-four clusters, corresponding to geographical areas sharing a common local authority in France, will be included. They will be randomly assigned to one of the following arms with a ratio of 1:1:1: a control group; a basic promotion group in which promotion of the StopBlues program will be done by local authorities; and an intensified promotion group in which basic promotion will be supplemented by an additional one in a general practitioner’s waiting room. The primary outcome measure will be the number of suicidal acts within each cluster over a 12-month period following the launch of the intervention. Baseline data will be collected for each cluster over the 12-month period prior to the trial. Secondary outcomes will include length of use of the StopBlues program, measures of help-seeking behaviors and level of psychological distress among users of the program, as well as the cost-effectiveness and budgetary impact of its promotion. A more sustained promotion by local authorities will also be implemented after 12 months in the control group and assessed using the same outcome measures.

**Discussion:**

This research should contribute to the sparse evidence base regarding the promotion of e-health programs and will support the wider delivery of the intervention evaluated if proven effective.

**Trial registration:**

ClinicalTrials.gov, ID: NCT03565562. Registered on 11 June 2018

## Background

Suicide is a human tragedy, resulting from significant psychological distress, and is among the leading causes of death worldwide [1]. France is one of the most affected Western European countries, with around 11,400 deaths by suicide (13.8 per 100,000 inhabitants) and nearly 200,000 suicide attempts annually [2–4]. Moreover, surveys in the general population have estimated that one out of 20 French people has attempted suicide at least once over their life course [5]. The societal costs of suicide are also particularly high. In countries where it has been documented, the magnitude of the economic burden of suicide has been found to range from several million to several billion dollars per year depending on the size of the population [6–9].

Yet, suicide represents a cause of avoidable death which could be significantly reduced by effective prevention programs in the general population targeting the main challenges in the field of suicide prevention. These challenges include impaired upstream identification and care for people at risk, whereas early detection and management of mental disorders and suicide ideation has been identified as one of the current key priorities for efficient suicide prevention by the World Health Organization (WHO) [10]. These observations underscore the need to develop tools to help self-recognition of suicidal risk and associated mental disorders in the general population, as well as to encourage help-seeking behaviors in this population. Given the high, and constantly increasing, rate of Internet access [11] and smartphone ownership [12], e-health programs could usefully complement current suicide-prevention measures in addressing the remaining challenges. It is estimated that an increasing share of the population resorts to the Internet in search of answers to their health concerns [13, 14], and this is particularly the case for mental health [15, 16]. It has also been shown that health-information-seeking on the Internet is higher among those with stigmatized health conditions, including depression and anxiety, and that users with these conditions increase their utilization of healthcare after accessing such information [17, 18].

The StopBlues program, including a smartphone application and an associated website, was developed in this context. This program is available free-of-charge for the general population living in France. Its content, accessible only to users aged over 18 years (as declared at the first connection), is evidence-based and was built based on previous literature reviews [19, 20] and on advice from expert groups, including people who have experienced psychological distress (participatory design process). All in all, the StopBlues program provides a comprehensive prevention approach including awareness strategies (provision of general information on mental health, stigma, benefits of help-seeking, etc.), screening strategies (mood-tracking and self-assessment questionnaires), strategies for accessing support (locator of nearby helps and emergency “get help now” button) and mental health strategies (safety plan and toolbox with relaxation and mindfulness exercises) as recommended by previous research [19].

However, e-health programs for suicide prevention should be associated with effective promotion strategies to ensure that people who could benefit from these programs are aware of them, as well as to help them identify reliable programs among those available online and encourage their use [19, 21]. In parallel with this targeted effect, such promotion campaigns may have ecological effects similar to those previously observed for other awareness campaigns [22–24], by decreasing the stigmatization associated with mental health issues in civil society, and changing the attitudes and norms associated with psychological distress in the adult general population. This would in turn facilitate help-seeking behaviors, leading to a decrease in the occurrence of psychological distress and suicidal ideations, and ultimately preventing suicidal acts. Suicide-prevention measures deployed in the community have been shown to be particularly effective in several studies [25–28], and previous research has involved cities or counties in the development of such measures [29, 30]. The additional involvement of health professionals, in particular general practitioners (GPs), in promotion campaigns for suicide prevention carried out at the local level could also be beneficial. Indeed, even if people with mental disorders prefer not to seek formal professional help [22], GPs are the health specialists whom people suffering from psychological distress or at risk of suicide most easily turn to, even if not always addressing these topics directly. Among people who committed suicide, 20% did so within 1 day of a visit to their GP [31]. Involving local authorities as well as GPs in promoting suicide-prevention actions through dedicated interventions could, therefore, have complementary benefits, provided that they address the potential lack of sufficient skills, as well as of technical, financial and human resources at their level. However, to the best of our knowledge, no study has involved a large number of local actors in the promotion of an evidence-based e-health tool for suicide prevention, although the development of promotion measures for e-health programs has been advocated by previous work [19, 21].

### Objectives of the trial

#### Primary objective

In this context, the primary objective of our trial (“*Programme de Recherche INTerventionnelle et Evaluative Mené pour la Prévention du Suicide*”, PRINTEMPS) is to assess the effectiveness of an intervention which includes a tailored, local-level promotion of the StopBlues program, and to compare the effectiveness of two types of local promotion (with or without a passive involvement of GPs through their waiting rooms) on deaths by suicide and suicide attempts over a 12-month period.

#### Secondary objectives

The secondary objectives will be measured both at the cluster level (geographical area sharing a common authority) and among users of the StopBlues program.

Secondary objectives considered at the cluster level are as follows:

1. To assess the cost-effectiveness and to run a budgetary impact analysis of the intervention

Secondary objectives measured among users of the StopBlues program are as follows:

2. To assess the effectiveness of the intervention in terms of the intensity of the utilization of the program
3. To assess the effectiveness of the intervention for help-seeking behaviors and participation in supportive activities (any activity that a user finds beneficial for their mental health)
4. To assess the effectiveness of the intervention for the evolution of the level of psychological impairment

It should be noted that an additional form of promotion (more sustained promotion by local authorities, described below) will be implemented after 12 months in the control group and evaluated using the same primary outcome measure. In parallel, the long-term effectiveness of the two main types of local promotion (with or without a passive involvement of GPs through their waiting room) will also be assessed.

## Methods/design

### Study design

This study will be implemented as a three-arm, parallel-group, cluster-randomized controlled superiority trial, with before-and-after observation. Cluster-randomization was deemed mandatory because of potential contamination issues. Indeed, the very nature of the intervention assessed (i.e., a promotion program) cannot be applied at the individual level. This is typically a cluster-cluster intervention, as defined by Eldridge et al. [32]. Clusters were defined as geographical areas sharing a common local authority. These local authorities can either be cities or a grouping of cities (“*communautés de communes*” or “*communautés d’agglomérations*”) based on the local health governance of each area. If several adjacent local authorities volunteer individually to participate in the trial, they will be considered as a unique local authority.

Clusters will be randomly assigned to one of the following three arms with a ratio of 1:1:1:

- Arm 1: no promotion of the StopBlues program (*control group*)
- Arm 2: promotion of the StopBlues program by local authorities only (*basic promotion group*)
- Arm 3: promotion of the StopBlues program by local authorities with an additional passive involvement of GPs through their waiting rooms (*intensified promotion group*)

The initial trial duration will be of 12 months. Baseline data on the number of suicidal acts, collected 12 months prior to the intervention, will also be considered in each cluster. After a 12-month post-randomization period, clusters from the control group will be allowed for a further 12-month period to launch their promotional campaign supported by the research team through regular contacts and additional technical and financial resources in comparison to arms 2 and 3 (*more sustained promotion by local authorities*). This will facilitate the recruitment of clusters as well as their adherence to the intervention during the first 12-month period [33]. It will also enable us to determine whether promotion led by local authorities could be improved by supporting them in the provision of additional promotional resources.

**Fig. 1:**
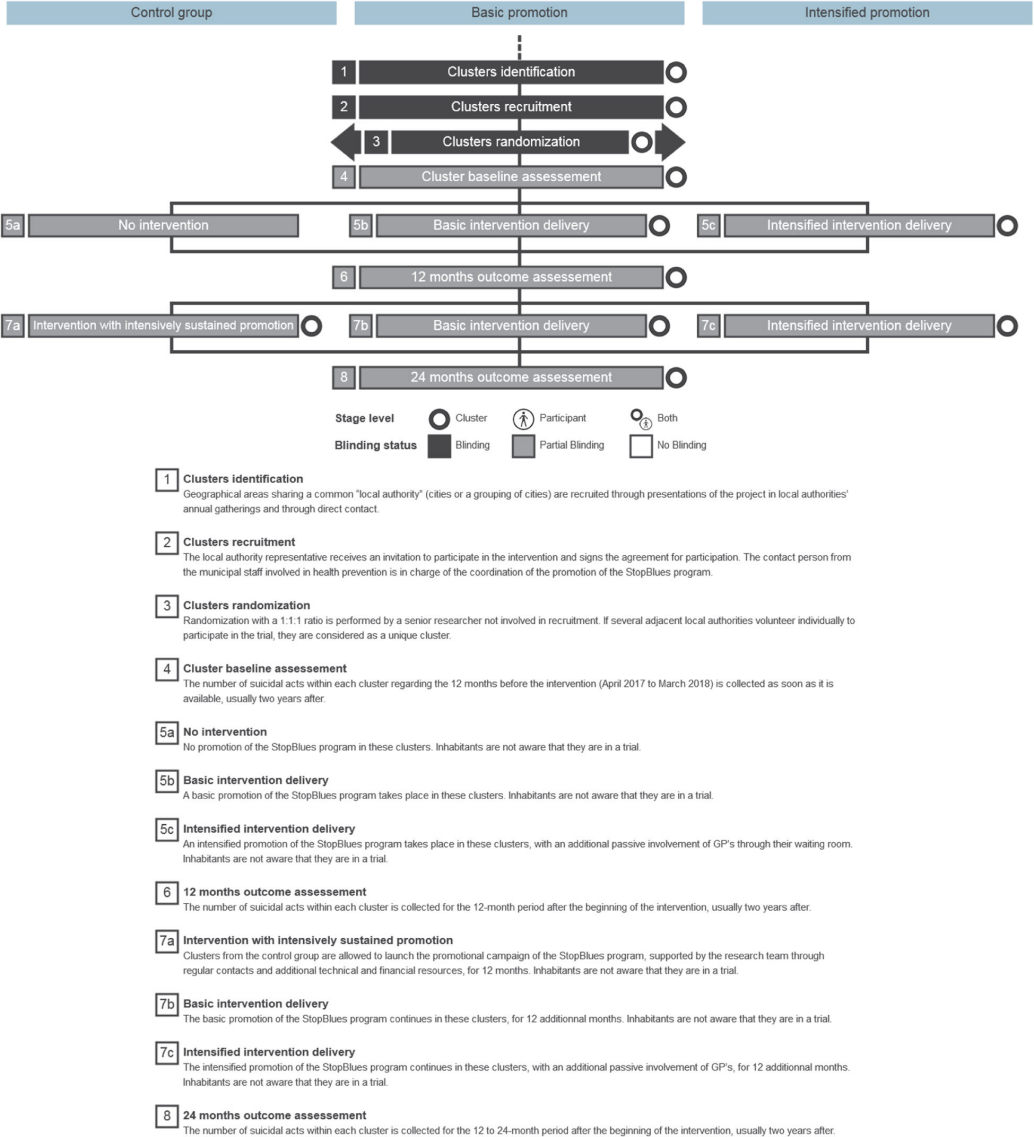
Timeline cluster tool of the intervention

A complete description of the different steps of the trial is presented in Fig. 1 using the Timeline cluster tool of Caille et al. [34].

We adhere to the Standard Protocol Items: Recommendations for Interventional Trials (SPIRIT) guidelines in reporting the trial in this protocol (see Supplementary material, Fig. 1 and Table 2). Items from the WHO Trial Registration Data Set (TRDS) [35, 36] are provided in Table 1.

**Table 1:** Items from the World Health Organization (WHO) trial registration data set

Data category	Information
Primary registry and trial identifying number	ClinicalTrials.gov, ID: NCT03565562
Date of registration in primary registry	11 June 2018
Secondary identifying numbers	Grant from the French Research Institute in Public Health: agreement 026/14
Source of monetary or material support	French Public Health Agency (*Santé Publique France*) in the frame of the primary prevention call for proposal of the French Research Institute in Public Health
Primary sponsor	Clinical Research Department, French National Institute of Health and Medical Research (INSERM), contact: promoteur.inserm@inserm.fr
Secondary sponsor	None
Contact for public queries	Pr Karine Chevreul (karine.chevreul@urc-eco.fr)
Contact for scientific queries	Pr Karine Chevreul (karine.chevreul@urc-eco.fr) and the PRINTEMPS team (printemps@urc-eco.fr)
Public title	*Programme de Recherche INTerventionnelle et Evaluative Mené pour la Prévention du Suicide* (PRINTEMPS)
Scientific title	A cluster-randomized controlled trial of the local promotion of a smartphone application and associated website for the prevention of suicidal behaviors in the adult general population in France
Country of recruitment	France
Health conditions or problem(s) studied	Suicide, depression, psychological distress
Intervention	Intervention 1: tailored promotion of the e-health StopBlues program at the cluster level without involving general practitioners. Intervention 2: tailored promotion of the e-health StopBlues program at the cluster level with passive involvement of general practitioners through their waiting room. Control intervention: no promotion of the StopBlues program.
Key inclusion and exclusion criteria	Clusters: geographical areas with volunteer local authorities located in France (mainland and overseas territories) which provided a signed agreement to participate Participants: all adults living in participating clusters
Study type	Interventional study with randomized allocation, parallel assignment and no masking for prevention purpose
Date of first enrollment	22 August 2018
Target sample size	34 clusters for a total of more than 4 million adult inhabitants
Recruitment status	On-going
Primary outcome	Number of suicidal acts at the cluster level during a 12-month period, including deaths by suicide and suicide attempts. Data will be extracted from administrative databases.
Key secondary outcomes	Number of deaths by suicide and suicide attempts during a 24-month period Cost-effectiveness and budgetary impact analysis: fixed costs for the development of the promotion tools, semi-fixed costs for the implementation of the intervention, costs of suicidal acts, level of health-related quality of life and associated utility (validated questionnaire). For participants who used the StopBlues program: Effect on the intensity of the utilization of the StopBlues program by its users: number of downloads and connections for each zip code corresponding to local authorities included in the trial, time-lapse between the registration and the last connection to the StopBlues program for each user, proportion of users who came to know the program through the different communication channels, proportion of users who registered for a relative and not for themselves, proportion of users who completed a safety plan. Effect on the help-seeking behaviors of users of the StopBlues program and their participation in supportive activities: help-seeking and participation in supportive activities collected with an ad hoc questionnaire adapted from a validated one. Effect on the level of psychological impairment of the users of the StopBlues program: level of psychological distress, depression, anxiety, suicidal risk (validated questionnaires) (see Table 2 for more details)
Ethics review	Approved by the Ethics Committee of the French National Institute for Health and Medical Research (Approval n° 15–240 of 7 of July 2015). Approved by the French Advisory Committee for Data Processing in Health Research (Approval n° 15–793 of 30 September 2015). Approved by the French Data Protection Authority (Decision DR-2016-421 of 3 November 2016)

### Recruitment and eligibility

Thirty-four clusters will be recruited in France, through presentations of the research project in local authorities’ annual gatherings and through direct contact from the research team. Included local authorities, located in France (either mainland France or overseas territories), will be all those that volunteer to participate in the intervention, and which provide a signed agreement for participation in the research. Their list will be available upon request to the research team.

The StopBlues program will be freely available in the general population and, therefore, accessible by the populations of all local authorities involved in the trial.

### Sample size and power

The number of clusters was fixed by the identification of 34 clusters that agreed to be involved in the trial. The proportion of inhabitants who died from suicide or who made suicide attempts over a 12-month period was estimated to be 0.215% (personal data). We used an expected rate of 0.190% in the experimental group, which represents a reduction of 11.6%, slightly higher than the 10% global target supported by WHO for interventions on suicide [10]. In a two-arm, individually randomized trial, this would lead to a required number of participants of 1,229,350 to achieve a power of 80% with a Type I error rate of 2.5%. Type I error is set at 2.5% because two experimental groups are compared to the control group. Clustering also has to be taken into account. As the number of voluntary clusters was fixed, we calculated what the intraclass correlation coefficient (ICC) would be in order to achieve a power of 80% with our pre-specified number of clusters. Although we acknowledge that such an approach is not classical, it enables us to take into account feasibility reasons. Our mean cluster size equals 138,172 inhabitants and, therefore, randomizing 11 clusters in each arm allows for achieving 80% power if the ICC is around 10^−5^. Although this is a very low ICC, one has to keep in mind that clusters are geographical areas and ICCs are known to be lower in large clusters as compared to small ones. Moreover, ICC is known to depend on prevalence [37], with small ICC values for prevalence close to 0 or 1, and we expect very low rates (about 0.2%). As a consequence, we considered that a 10^−5^ ICC value would be appropriate in our trial.

### Randomization

Clusters will be randomized all at once and their allocation will be carried out using stratified randomization by minimization. The minimization will use two important factors that can affect the statistical modeling and population homogeneity in the three arms. Indeed, minimization enables a better balance of the prognostic parameters between the different strategy groups than classical randomization, especially when the number of randomization units is small. The considered factors will be the type of local authority forming the cluster (independent cities only, cities which have been grouped for the present study because they were adjacent, or groups of cities, i.e. “*communautés de communes*” or “*communautés d’agglomérations*,” which are administratively grouped) and the size of the population, in order to have close distribution of local authority types and median size of population in the three arms.

The minimization parameters will be used to adjust statistical analyses of the primary and secondary outcomes. Randomization will be performed by an independent statistician who will not be involved in the further steps of the research.

### Blinding

The very nature of the intervention does not allow any form of blinding. However, participants living in the selected clusters will not be informed that their local authority is included in a randomized trial. Only those who use the StopBlues program will be asked to provide consent to have their data collected through the application or associated website (but this does not correspond to a consent to be included in the randomized trial). Moreover, the primary outcome will be assessed using administrative databases with a pre-specified algorithm. Therefore, although there is no blinding in the present trial, it is not at risk of performance bias or detection bias.

### Intervention to be evaluated

The intervention evaluated corresponds to the tailored local promotion of the StopBlues program. Its description below complies with the Template for Intervention Description and Replication (TIDieR) Checklist for intervention description [38].

The StopBlues program will be promoted by the French local authorities participating in the trial. A basic communication toolkit, including a promotion guide, posters, leaflets and key communications messages, will be provided to each local authority at the beginning of the intervention. In addition, a contact person from the municipal staff already involved in health prevention will be appointed in each local authority. They will be in charge of coordinating the promotion of the StopBlues program at the local level and will participate in a day-long training session prior to the beginning of the promotion campaign. Local authorities will then be in charge of promoting the program by choosing their usual municipal communication medium (local newspapers, bulletins and billboards, posters in local shops and bus stops, etc.), by involving local actors of their choice and by developing additional promotion tools if they wish. They will, therefore, be able to develop a tailored intervention adapted to their local context.

Additional promotion of the StopBlues program (intensified promotion) will be carried out in several local authorities by the additional involvement of local GPs. As GPs’ workload is significant in France, due to the fact that they have been put at the heart of care coordination [39], a passive involvement of GPs in the promotion of the StopBlues program was designed. They will receive communication tools on the StopBlues program, such as leaflets or posters, to put in their waiting rooms in order to inform patients and their relatives about the existence of the program.

Finally, the local authorities of the control group will be offered a more sustained promotion at 12 months, which is not part of the main intervention but will also be evaluated during the trial. This promotion will consist of the same promotion as in the basic promotion group to which will be added regular support from the research team with stronger contacts and technical and financial resources for local authorities to implement the promotion and print the tools (flyers, posters, etc.). The inclusion of GPs in this promotion process will be left to local authorities’ discretion.

### Outcomes

#### Primary outcome measure

The primary outcome measure will be the number of suicidal acts (both death by suicides and suicide attempts) during the 12 months after the start of the intervention. The number of deaths by suicide will be obtained from the Epidemiology Center for Medical Causes of Death (“*Centre d’épidémiologie sur les causes médicales de décès”*, CépiDc) while the number of suicide attempts will be approximated by the number of suicide attempts in contact with the healthcare system (other than primary care only). To estimate the number of suicide attempts seen in acute care, we will use the exhaustive French National Hospital Discharge Database (“*Programme de médicalisation des systèmes d’information en médecine, chirurgie, obstétrique*,*”* PMSI-MCO) [40]. To estimate the number of suicide attempts seen in emergency care, we will use the *Oscour* database which records visits to emergency departments [41]. Participants from clusters included in the study will be identified through their zip code of residence.

#### Secondary outcome measures

##### Cost-effectiveness and budgetary impact analysis

The cost-effectiveness and budget impact of the intervention will be assessed using a societal perspective. The budget impact analysis will be carried out at 12 and 24 months while the cost-effectiveness analysis will be evaluated over the entire user’s lifetime.

Costs linked to the implementation of the intervention will be measured at 12 months and at the end of the intervention. They will include fixed costs resulting from the creation of the promotion toolkit for the StopBlues program. They will also include semi-fixed costs, linked to potential specific communication strategies developed by the different local authorities for the implementation of a tailored intervention, and to the human resource costs of involving municipal staff. Costs of suicidal acts will be estimated by considering direct costs for the healthcare system as well as indirect costs for individuals attempting suicide and their relatives.

Moreover, we will use the 12-item Short Form health survey (SF-12) [42] among users of the StopBlues program to obtain a generic measure of perceived health-related quality of life which can then be used to estimate a preference-based measure of health for economic evaluations [43]. This has been previously validated in French [44].

##### Intensity of the utilization of the StopBlues program by its users

The intensity of the utilization of the StopBlues program by its users will be assessed through a complementary set of measures. They include the number of downloads and connections to the StopBlues program, the time-lapse between the registration and the last connection to the program; the proportion of users who were made aware of the program through the different communication channels (such as communication by their local authority, a relative, GP’s waiting room, etc.) and the proportion of users who subscribed for a relative and not for themselves. The last two measures will be assessed at the first connection to the StopBlues program (T0) by an ad hoc questionnaire. Finally, we will measure the proportion of users who completed a safety plan at 12 and 24 months (Table 2).

**Table 2:** Measures, data collection methods and timeframe

Measure	Data collection method	Periodicity of collection^**a**^							
		M0		M1	M2	M3	M6	M12	M24
*Outcomes measured at the cluster level*									
Number of deaths by suicide and suicide attempts	Database of the epidemiology center for medical causes of deaths for deaths by suicide and extraction from the French National Hospital Discharge Database and the *Oscour* database recording visits to emergency departments for suicide attempts	X						X	X
Fixed costs for the development of the promotion tools and semi-fixed costs for the implementation of the tailored intervention	Data collected by the research team and local authorities							X	X
*Outcomes measured among users of the StopBlues program*									
		**T0 or M0**	**T0’**^b^	**T1**	**T2**	**T3**	**T6**	**T12** **or** **M12**	**T24 or M24**
Number of downloads and connections	Software analytics							X	X
Length of use (time-lapse between the registration and the last connection)	Software analytics							X	X
Proportion of users hearing about the StopBlues program through the different communication channels	Ad hoc questionnaire	X							
Proportion of users who registered for a relative and not for themselves	Ad hoc questionnaire	X							
Proportion of users who completed a safety plan	Software analytics							X	X
Level of psychological distress	GHQ-12	X		X	X	X	X	X	X
Level of depression^c^	PHQ-9	X		X	X	X	X	X	X
Level of anxiety^c^	GAD-7	X		X	X	X	X	X	X
Level of suicidal risk^b^	MINI suicidality module		X	X	X	X	X	X	X
Level of a relative’s depression (for users coming for a relative and not for themselves)	MADRS	X							
Level of health-related quality of life	SF-12	X		X	X	X	X	X	X
Help-seeking behaviors and participation in supportive activities	Ad hoc questionnaire adapted from the General Help-seeking Questionnaire (GHSQ)	X		X	X	X	X	X	X

*GAD-7* 7-item General Anxiety Disorder, *GHQ-12* 12-item General Health Questionnaire, *MADRS* Montgomery-Asberg Depression Scale*, PHQ-9* 9-item Patient Health Questionnaire*, SF-12* 12-item Short Form health survey

^a^M0, M1, etc. refer to the time-lapse in month to the launch of the intervention while T0, T1, etc. refer to the time-lapse in month to the first connection of the user to the StopBlues program

^b^T0’ corresponds to the first time that the MINI suicidality module is submitted to the user (this only happens if the user has a bad score at the PHQ-9 and/or at the GAD-7 and/or for the Likert scales of the mood-tracking system which are not used in the evaluation) so T0’ can, therefore, differ from T0. In the case of the level of suicidal risk, T1, T2, T3, T6, T12 and T24, therefore, corresponds to the time after T0’ and not T0

^c^Depression and anxiety will be measured if the GHQ-12 score suggests potential psychological distress

##### Help-seeking behaviors and participation in supportive activities by users of the StopBlues program

Help-seeking behaviors (towards health professionals or lay individuals) and participation in supportive activities by users of the StopBlues program will be assessed using an ad hoc questionnaire adapted from the General Help-seeking Questionnaire (GHSQ) [45] at users’ first connection (T0) and then regularly until the end of the intervention (Table 2).

##### Level of psychological impairment of users of the StopBlues program

Several outcome measures will be considered to assess the effect of the intervention on the level of psychological impairment of users of the StopBlues program. They include psychological distress, level of depression, anxiety and suicidal risk (including a past history of suicide attempts). They will be measured using the 12-item General Health Questionnaire (GHQ-12) for psychological distress [46], the Patient Health Questionnaire (PHQ-9) for the level of depression [47], the seven-item Generalized Anxiety Disorder-7 (GAD-7) for the level of anxiety [48] and the Mini International Neuropsychiatric Interview (MINI) suicidality module for the level of suicidal risk [49]. All questionnaires have previously been used in the French language and are estimated to have good measurement properties [50–59]. The level of psychological distress will be measured at the first connection of the user to the StopBlues program (T0) and then regularly until the end of the intervention (see Table 2). Depression and anxiety levels will be measured for users having a GHQ-12 score suggesting potential psychological distress. Users will also have to indicate their overall mood level through a mood-tracking system including three simple Likert scales ranging from 0 (worst possible) to 100% (perfect). These will focus respectively on users’ global health, mood and overall level of energy. These scales will not be considered in the evaluation of the level of psychological impairment of users of the StopBlues program but will condition the measurement of suicidal risk. The suicidal risk will indeed only be measured if the score of the PHQ-9 questionnaire is superior or equal to 10, or if the score of the GAD-7 questionnaire is superior to 7, or if the score on one of the scales of the mood-tracking system is inferior or equal to 40% or inferior or equal to 50% with a 20% decrease in comparison to the previous score. The suicidal risk will be measured at T0’ (see Table 2) and then regularly until the end of the intervention. Where the user has registered with the StopBlues program for a relative and not for themselves, they will be asked to fill in the Montgomery-Asberg Depression Scale (MADRS) [60] at the first connection (T0).

#### Long-term effectiveness of the intervention

The long-term effectiveness of the intervention will be measured by the numbers of deaths by suicide and suicide attempts (in contact with the healthcare system other than primary care only) considered separately during the 24 months following the start of the intervention.

#### Effectiveness of the more sustained promotion by local authorities

The effectiveness of the more sustained promotion by local authorities will be measured by the numbers of deaths by suicide and suicide attempts considered separately in the 12 months following the start of this form of promotion in the control group.

Table 2 provides a summary of all outcome measures collected by level of availability as well as their timeframe.

If users of the StopBlues program stop connecting to it, they will receive push-up notifications on their smartphones or reminder emails regarding the completion of questionnaires, to improve adherence.

#### Statistical considerations

Characteristics of local authorities and of users of the StopBlues program will be described by the mean and standard deviation (SD) for continuous variables or by number (%) for categorical variables.

To assess the effectiveness of the intervention on the primary outcome (number of suicidal acts at the local authority level), we will compare the number of overall suicidal acts at 12 months and of suicides and suicide attempts separately between the trial arms adjusted on the number observed during the 12 months before the intervention.

We will use a Poisson regression analysis with clusters as analysis units and cluster size as offsets and adjust for baseline data and local authorities’ characteristics (population size, ecological index of deprivation, type of local authorities forming the cluster, etc.). A similar method will be used for all secondary outcomes measured at the level of local authorities.

For secondary outcomes measured on the users of the StopBlues program, mixed-model repeated-measures analyses will be used. This method will enable the inclusion of all users regardless of their level of adherence to the program, which will allow us to carry out the analysis despite some missing values. Adjustments will be made on the initial level of psychological distress of each user and on demographics. By using mixed-model repeated-measures analyses [61], we will also be able to account for potential clustering effects through the incorporation of adequate random effects for local authorities. Transformation of outcome variables will be done as necessary to meet distributional assumptions and to accommodate potential outlying observations. Additional subgroup analyses will be carried out to assess separately the effect of the intervention on users with a history of suicide attempts and those without.

#### Ethical considerations

This trial was granted ethical approval by the relevant ethics committee (see the “Declarations” section below). It should be noted that participants are not informed that they are included in a randomized trial. Randomization is carried out at the cluster level (geographical areas). There are no opt-out options for people living in geographical areas allocated to one of the two experimental groups, and there is no direct relationship with participants to collect data for the primary outcome. Absence of information and consent to be included in the trial is, therefore, in agreement with the Ottawa Statement on ethical issues for cluster-randomized trials [62]. This was also positively assessed by the relevant ethics committee. In addition, the risk to users of the StopBlues program was evaluated as minimal due to a number of specific features of the program (emergency “get help now” button, safety plan, locator of nearby helps, push-up notifications reminding users to get help, information on prevention regularly updated, etc.). In particular, if someone expressed suicidal ideation while using the program, they would straight away be directed to a call either to general health emergency services (“get help now” button) or to their personal emergency contacts (professionals or relatives) if they filled in the safety plan. All this explains that no data monitoring committee was created for this study and that no detailed stopping guidelines for the trial were enacted. Nevertheless, if an increase in the number of suicidal acts was to be found in the participating local authorities during the first round of analysis of data, the decision to stop the trial could be taken in consultation with funders and health authorities. They will also be involved in the decision to continue the intervention after the trial based on its results.

All users of the StopBlues program will provide written informed consent before their data is collected for research purpose. They will indeed have to sign a consent form for participation in the intervention and will be able to withdraw from the research whenever they want. Their data will be stored on a secure server and any identifying information necessary for the access to the program (email address) will be encrypted through a unique user identifier. Data files used for linkage will be stored separately from raw research data.

It should be noted that both local authorities included in the trial and users of the StopBlues program will not be prohibited from participating in other interventions and/or receiving concomitant care.

#### Dissemination plan

Findings from the research will be published in aggregated form at the local authority level (no information on individual users of the StopBlues program will ever be provided). Given the broad scope of research objectives for this trial, the dissemination plan for this study includes several publications reporting on preliminary and secondary outcomes. Authorship will be based on actual contributions to the research work. Scientific publications will be complemented by communications aimed at policy-makers in order to ensure the continuation of the intervention, after termination of its funding in the framework of the research project, if results prove satisfactory.

The trial outcomes will be reported in line with the Consolidated Standards of Reporting Trials (CONSORT) guidelines for cluster-randomized trials [63, 64]. An anonymized dataset (therefore, not requiring additional consent) will also be made available by the authors within 3 years of the final collection of data. This dataset will be made available to other researchers for the development of further studies, upon submission of a research protocol to our research consortium and after obtaining adequate ethical approvals.

## Discussion

Previous research has shown that awareness campaigns in the community can be effective for suicide prevention, by decreasing the stigma associated with mental disorders and help-seeking, in particular when such campaigns are associated with other interventions [22, 65]. However, so far, there is a dearth of research regarding promotion of e-health tools for suicide prevention. The intervention evaluated in the PRINTEMPS study is, therefore, innovative. In addition, formal and rigorous evaluation of this type of intervention in the general population is lacking in the current literature. We will assess the effectiveness of the intervention through the implementation of a three-arm, parallel-group, cluster-randomized controlled trial, which will add to the currently sparse evidence base, both for the promotion of e-health programs and the involvement of the general population.

If proven effective, the intervention of the PRINTEMPS study could be more widely delivered in French-speaking settings, providing a valuable new resource for suicide prevention. In parallel, the extrapolation of the promotion strategy to other contexts could be explored. This includes extrapolation to other national contexts; for example, by adapting it for use in several other European countries, and to other specific populations such as, for example, an e-health suicide-prevention tool for younger individuals or individuals experiencing mental health issues in the workplace. While participating local authorities are selected on a voluntary basis and cannot be expected to be fully representative of all French local authorities, replication and transferability will be facilitated by a process evaluation of the PRINTEMPS study, which will complement the assessment of the effectiveness of the intervention by assessing the fidelity and the quality of the implementation. Qualitative methods will be used to clarify causal mechanisms and identify the contextual factors possibly influencing them [66], and to identify the key functions of the intervention [67–69]. Using interviews with the contact person in charge of managing the implementation of the intervention in the different clusters and an ethnographic case study in a reasoned sample of local authorities, this evaluation should highlight the conditions in which the intervention is effective, and will look for its possible unexpected effects.

## Trial status

The protocol reported here corresponds to the version of 1 September 2018. The intervention of the PRINTEMPS study was launched in April 2018 and the recruitment of potential users of the StopBlues program is taking place until March 2020.

## Supplementary information

**Additional file 1.** Standard Protocol Items: Recommendations for Interventional Trials (SPIRIT) Checklist.

## Data Availability

The datasets generated and/or analyzed during the current study will be made available from the corresponding author (unique handler of the final dataset) on reasonable request within 3 years of the final collection of data.

## Abbreviations

CCTIRS: French Advisory Committee for Data Processing in Health Research
CépiDc: Epidemiology Center on Medical Causes of Death
CNIL: French Data Protection Authority
CONSORT: Consolidated Standards of Reporting Trials
GAD-7: Generalized Anxiety Disorder-7
GHQ-12: 12-item General Health Questionnaire
GHSQ: General Help-seeking Questionnaire
GP: General practitioner
ICC: Intraclass correlation coefficient
INSERM: French National Institute for Health and Medical Research
MADRS: Montgomery-Asberg Depression Scale
MINI: Mini International Neuropsychiatric Interview
PHQ-9: Depression module of the Patient Health Questionnaire
PMSI-MCO: French National Hospital Discharge Database
PRINTEMPS: *Programme de Recherche INTerventionnelle et Evaluative Mené pour la Prévention du Suicide*
SD: Standard deviation
SF-12: 12-item Short Form health survey
SPIRIT: Standard Protocol Items: Recommendations for Interventional Trials
TIDieR: Template for Intervention Description and Replication
TRDS: Trial Registration Data Set
WHO: World Health Organization
